# Quaternion Entropy for Analysis of Gait Data

**DOI:** 10.3390/e21010079

**Published:** 2019-01-17

**Authors:** Agnieszka Szczęsna

**Affiliations:** Institute of Informatics, Silesian University of Technology, Akademicka 16, 44-100 Gliwice, Poland; agnieszka.szczesna@polsl.pl

**Keywords:** quaternion entropy, approximate entropy, gait data, motion data analysis

## Abstract

Nonlinear dynamical analysis is a powerful approach to understanding biological systems. One of the most used metrics of system complexities is the Kolmogorov entropy. Long input signals without noise are required for the calculation, which are very hard to obtain in real situations. Techniques allowing the estimation of entropy directly from time signals are statistics like approximate and sample entropy. Based on that, the new measurement for quaternion signal is introduced. This work presents an example of application of a nonlinear time series analysis by using the new quaternion, approximate entropy to analyse human gait kinematic data. The quaternion entropy was applied to analyse the quaternion signal which represents the segments orientations in time during the human gait. The research was aimed at the assessment of the influence of both walking speed and ground slope on the gait control during treadmill walking. Gait data was obtained by the optical motion capture system.

## 1. Introduction

The parameters associated with chaos are measures of dimension, rate of information (entropy) and the Lyapunov determinant. Kolmogorov entropy *K* is known as a chaos metrics and the value of entropy can be used for the classification of underling dynamic systems [1]. The connection of Kolmogorov entropy and Lyapunov determinants of the system is defined by Pessin’s theorem. Sensitive dependence on initial conditions is a distinguishing feature of chaotic behavior. Initially, arbitrarily close points in the phase space produce significantly different trajectories. Characteristically, trajectories in chaotic systems diverge exponentially and Lyapunov exponents (LLE) were proved to be a good quantitative measure for the average rate of exponential divergence of two trajectories. Hence, positive LLE is indicative of unpredictable behavior. *K* is basically equal to the sum of the positive LLE of the system.

Theoretical defined entropy *K* is not able to be obtained on a base on finite, noisy data (signals) obtained from real measurements. The amount of data typically required to archive convergence is between $10^{d}$ to $30^{d}$ points to fill out a d-dimensional strange attractor for the chaotic system [2]. To overcome this, the approximate entropy, as a family of statistics, has been proposed [3]. It was shown that it can potentially distinguish low dimensional deterministic systems, periods and multiply periodic, high dimensional chaotic, stochastic and mixed systems [4]. The approximate entropy statistics is based on an Eckmann–Ruelle entropy formula [5], for the physical invariant measure for use with time series data.

This work presents the new quaternion, approximate entropy and its application to analysis of human gait kinematic data represented as a quaternion signal. This signal represents the segments orientations in time during the human gait. The research was aimed at the assessment of the influence of both walking speed and ground slope on the proposed quaternion entropy during treadmill walking. Gait data was obtained by the optical motion capture system. The analysis was carried out for femur, tibia and foot segments.

## 2. Materials and Methods

### 2.1. Background

Letting $q_{1},q_{2},\cdots ,q_{N}$ be a time series signal of measurements equally spaced in time, the sequence of vectors in $R^{m}$ can be defined as x(1),x(2),⋯,x(N−m+1), where $x\left(i\right)=\left[q_{i},q_{i+1},\cdots ,q_{\left(i+m-1\right)}\right]$. Next, for each *i* and *j*, 1≤i≤N−m+1, and 1≤j≤N−m+1, the following function is defined as

(1) $$C_{i}^{m}\left(r,N\right)=\frac{\#(d\left[x(i),x(j)\right]\leq r)}{\left(N-m+1\right)},$$

where # is the number of elements in set, *r* is vector comparison distance and *m* is the dimension of created and compared vectors. The distance *d* is defined as follows:

(2) $$d\left[x(i),x(j)\right]=\max_{k=1,2,\cdots ,m}\left(\left|q_{i+k-1},q_{j+k-1}\right|\right).$$

The definitions above are based on correlation dimension derivation and are needed to define $\Phi^{m}\left(r\right)$ as

(3) $$\Phi^{m}\left(r,N\right)={\left(N-m+1\right)}^{-1}\sum_{i=1}^{N-m+1}\log\left(C_{i}^{m}\left(r,N\right)\right).$$

After fixing *m* and *r*, we obtain a definition of approximate entropy [3]:

(4) $$\mathrm{ApEn}\left(m,r\right)=\lim_{N\to \infty }\left[\Phi^{m}\left(r,N\right)-\Phi^{m+1}\left(r,N\right)\right].$$

Given *N* samples, we can define the following formula as statistics:

(5) $$\mathrm{ApEn}\left(m,r,N\right)=\Phi^{m}\left(r,N\right)-\Phi^{m+1}\left(r,N\right).$$

Informally, given *N* signal’s samples, the family of statistics ApEn(m,r,N) is a measure of probability that two sequences that are similar for *m* points remain similar, that is, within a tolerance *r*, in the next sample in a signal. Thus, a low value of ApEn reflects a high degree of regularity. Following family statistics, ApEn has been widely used in clinical cardiovascular studies [6,7,8,9] and neurology, among others, for analysis of electroencephalogram signals [10,11,12,13].

Similar derivation has related to complexity measure sample entropy (SampEn) [6]. Sample entropy in counting the similar vectors does not count self-matches. The ApEn entropy algorithm counts each sequence as matching itself, which is a way to avoid occurrence of log(0) in calculations. In practice, this causes some bias value in results. This was a very detailed discussion in [6,14]. However, ApEn and SampEn also indicate more self-similarity in the time series.

Entropy to gait analysis was used only for times series consisting of spatio-temporal parameters like step time, length and width, stride intervals [14,15] or segment trajectories [16]. Analysis of center of COP (center of pressure) trajectory by sample entropy can be found in [17]. In [18], multiscale entropy [19] was applied on trunk acceleration data collected during a gait of subjects of different ages: toddlers, pre-scholar and scholar children, adolescents, young adults, adults and elderlies. Control entropy was used to analyse the acceleration data $a_{i}=\left[a_{x,i},a_{y,i},a_{z,i}\right]$ as three independent signals [20]. Each cited entropy used to analyse gait data is based on ApEnt formula.

Based on actual knowledge, there is no proposition to compute entropy to analyse gait data based on unit quaternions’ time series. This conception allows for processing correlated data and to obtain results taking into account rotations in 3D. The same preliminary studies were done based on quaternion energy and entropy to the classification of people based on gait data [21].

**Figure 1:**
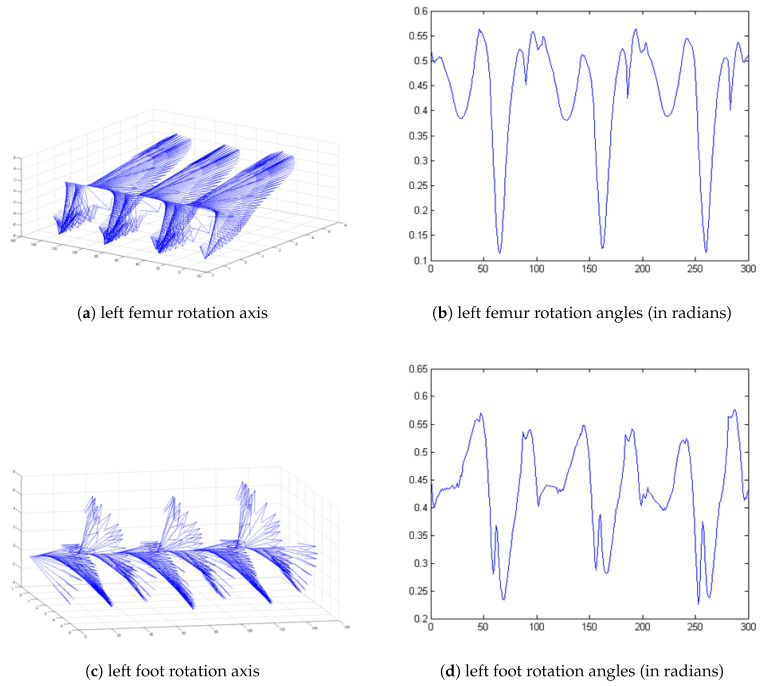
Axis and angle of rotation of femur and foot segments during gait (three strides).

In [22], the authors describe an example of application of a nonlinear time series analysis directed at identifying the presence of deterministic chaos in human gait kinematic data by means of the largest Lyapunov exponent (LLE). A positive LLE value is interpreted as an indicator of local instability. However, the LLE was computed based on separate Euler angles (three rotations angles about the axes of a coordinate system) or only angles of rotation in quaternion representation. Such analysis in quaternion form did not consider axis of rotation. In this research experiment, data from this same trial was used so the results will be compared. Figure 1a,c present the axis of rotation (imaginary part of quaternion) during 300 samples of gait (about 3 strides). Figure 1b,d show the angle of rotation (scalar part of quaternion). To analyse all aspects of movement during gait, all of the information should be processed. The new propositions of quaternion motion analysis tools are widely discussed in [23,24,25,26].

The *o* orientation indicates the orientation of the rigid body defined in the reference system (also named as the reference frame). Rotation means a change in orientation, $o_{1}\to o_{2}$. The group SO(3) (*special orthogonal group*) contains all rotations around the origin of the Euclidean coordinate system $R^{3}$. Rotations can be described by orthogonal matrices. Rotations can also be written as a combination of three Euler angles around the three coordinate system axes. The angles are usually referred to as *roll*, *pitch* and *yaw*. In this representation, the order in which the rotation is performed is important. There is also the phenomenon of blocking one degree of freedom of movement (*gimbal lock*).

In computer graphics, the most common description of rotation is unit quaternion [27,28]. Quaternions [27,28] are an extension of complex numbers, q∈H where H is a quaternion algebra. Quaternion $q=q_{0}+i\cdot q_{1}+j\cdot q_{2}+k\cdot q_{3}$, consists of real $q_{0}$ and imaginary part $i^{2}+j^{2}+k^{2}=i\cdot j\cdot k=-1$. Multiplication of two quaternions is marked as ⊗. We can write quaternion as vector and scalar part: $q=\left[q_{0},\vec{u}\right],\vec{u}=\left[q_{1},q_{2},q_{3}\right]$. Using the Euler rule for complex numbers, we have a rotation around the axis $\vec{u}$ by an angle α:

(6) $$q=\left(\cos\left(\frac{\alpha }{2}\right)+\vec{u}\cdot \sin\left(\frac{\alpha }{2}\right)\right).$$

In order to represent the rotation, unit quaternions are used, which fulfill the condition 〈q,q〉=1, where 〈.〉 is a scalar product. Unit quaternions lie on the hypersphere (denoted as $H_{1}$) embedded in Euclidean space 4D. The $H_{1}$ space is a SO(3) map with double mapping. This means that each rotation (or orientation) can be represented by two unit quaternions, called antipode. They represent a rotation around this same axis, but with a positive or negative angle. Conjunction quaternion represents inverse rotation $q^{*}$, which for unit quaternions is equal to inverse quaternion $q^{-1}$.

In summary, unit quaternions are a suitable mathematical tool for describing orientation and rotation in 3D space. It is possible to describe the rotation composition as multiplying quaternions. Conjunction quaternion represents inverse rotation. Such a description is free from the phenomenon of blocking one degree of freedom. The distance metrics between quaternions [29,30] have also been defined.

Using quaternions and building analysis in the domain $H_{1}$, we can process rotational data as correlated data with simultaneous rotation information around each axis of rotation. It also eliminates the problem of blocking one degree of freedom (*gimbal lock*) and allows a consistent mathematical record.

### 2.2. Quaternion Approximate Entropy

Let us assume that input a motion signal consists of quaternions: $q_{1},q_{2},\ldots ,q_{n}$ where $q_{i}\in H_{1}$ and $n=2^{k}$ for some k∈N.

Furthermore, the signal is processed by the selective negation (hemispherization), that is, every quaternion $q_{i}$$\left(i>1\right)$ is converted to $-q_{i}$ if $\langle q_{i},q_{i-1}\rangle <0$ due to duality of unit quaternions which represent rotations. It satisfies the requirement according to which two adjacent quaternions are located on the same hemisphere.

The new proposed metric, the quaternion, approximate entropy ApQuatEn, is based on Equation (5). Quaternions are of unit length, which means they are located only on a hypersphere $H_{1}$. Thus, to compare rotations, it is sufficient to calculate cosine distances between related quaternions, which is reflected by angles between vectors formed by quaternions’ components. The scalar product $\langle q_{1},q_{2}\rangle$ can be used to accomplish the task:

(7) $$d_{\cos\mathrm{ine}}\left(q_{i},q_{j}\right)=\frac{1-\langle q_{i},q_{j}\rangle }{2}.$$

The distance between two quaternions $\left|q_{i+k-1},q_{j+k-1}\right|$ in Equation (2) is defined as $d_{\cos\mathrm{ine}}$, so the equation is:

(8) $$d\left[x(i),x(j)\right]=\max_{k=1,2,\cdots ,m}\left(d_{\cos\mathrm{ine}}\left(q_{i+k-1},q_{j+k-1}\right)\right).$$

### 2.3. Treadmill Experiments

Time series were extracted from treadmill gait sequences which were recorded in the Human Motion Laboratory (HML) of the Polish-Japanese Academy of Information Technology (Bytom, Poland) by the optical motion capture system. Data was recorded with the use of the Vicon Nexsus optical motion capture system (Vicon Motion Systems Ltd., Oxford, UK). The skeleton model of 22 segments is applied and positions are traced based on 39 markers in standard full body Plug-In Gait marker placement. For analysis, only Euler angles’ orientations of femur, tibia, foot (left and right) segments were converted to unit quaternions time series signal.

Application of the AC5000M treadmill (SCIFIT Corporate Headquarters, United States) allowed recordings in three variants: at the preferred walking speed (PWS) of each subject (denoted as *Normal* speed), at 80% of the PWS (denoted as *Slower* speed) and at 120% of the PWS (denoted as *Faster* speed). Additionally, the recordings with the PWS on an inclined treadmill at slope of +7deg (denoted as slope *Up*) and −3deg (denoted as slope *Down*). Three sequences of continuous walking of lengths of several dozen seconds were recorded with a frequency of 100 Hz at a given walking speed for every person. In this way, the total number of sequences recorded for every subject was equal to 15. At any time, participants could take a rest upon request. The mean duration of a sequence was equal to 71.9 s with standard deviation of 10.7. A sequence consisted in a mean of 57.8 strides with standard deviation of 5.3. Seventeen healthy subjects (6 women and 11 men) participated in the experiments. Mean values of their age 27.18 (standard deviation 8.72), height 174.47 cm (standard deviation 6.46), weight 71.14 (standard deviation 10.06) and preferred walking speed 2.38 m/s (standard deviation 0.63). A stride interval (i.e., the time elapsed between consecutive ipsilateral heel strikes) varied across subjects and variants of the walking speed and treadmill slope. Mean values of a stride interval for every variant are 1.20 s for *Normal* speed (standard deviation 0.15), 1.40 m/s for *Slower* speed (standard deviation 0.21), 1.13 m/s for *Faster* speed (standard deviation 0.14), 1.27 m/s for *Slope-up* (standard deviation 0.18) and 1.24 m/s for *Slope-down* (standard deviation 0.16). This same dataset was also used in [22,31].

## 3. Results and Discussion

For all time series, data of 17 participants in each configuration (*Normal*, *Slower*, *Faster*, *Up*, *Down*) repeated 3 times gives 255 data streams. For all data streams, the ApQuatEn was calculated. The following length of vectors *m* are used in calculations: 2,3,4. In addition, the value of threshold *r* has to be defined. The proposition is to use the mean value of distance $d_{\mathrm{cosine}}$ between each following $q_{i}$ and $q_{i+1}$ quaternion in time series. The *r* value was calculated for each sequence (each segment and each experiment configuration). All computations were performed using Matlab software (2016a, MathWorks Inc., Natick, MA, USA).

**Figure 2:**
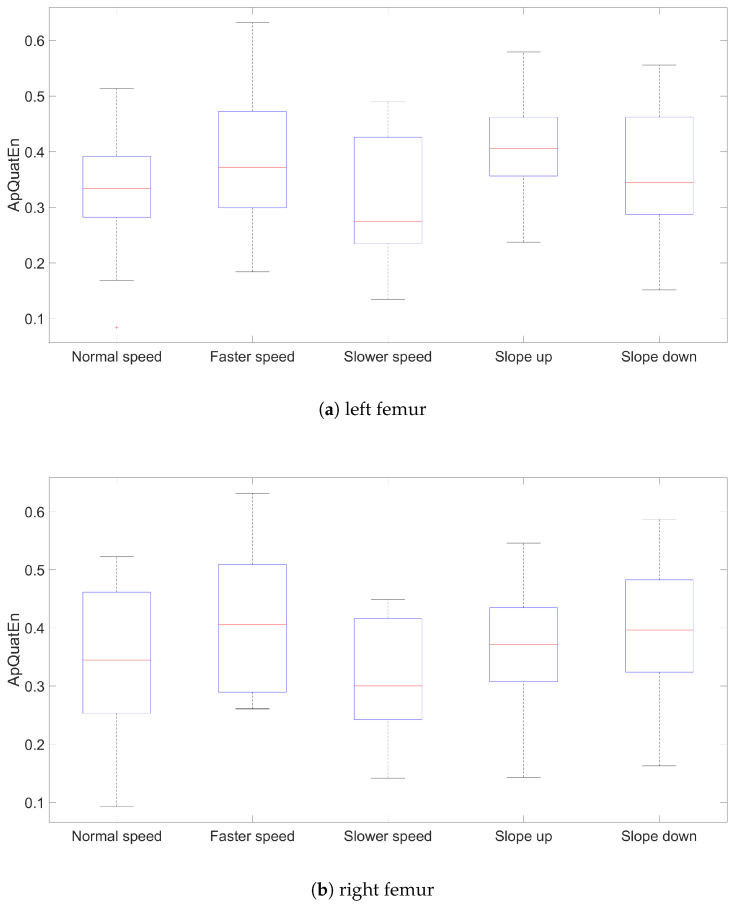
Results values of ApQuatEn (m=2, $r=\mathrm{mean}(d_{\mathrm{cosine}})$) for left and right femur segments.

**Figure 3:**
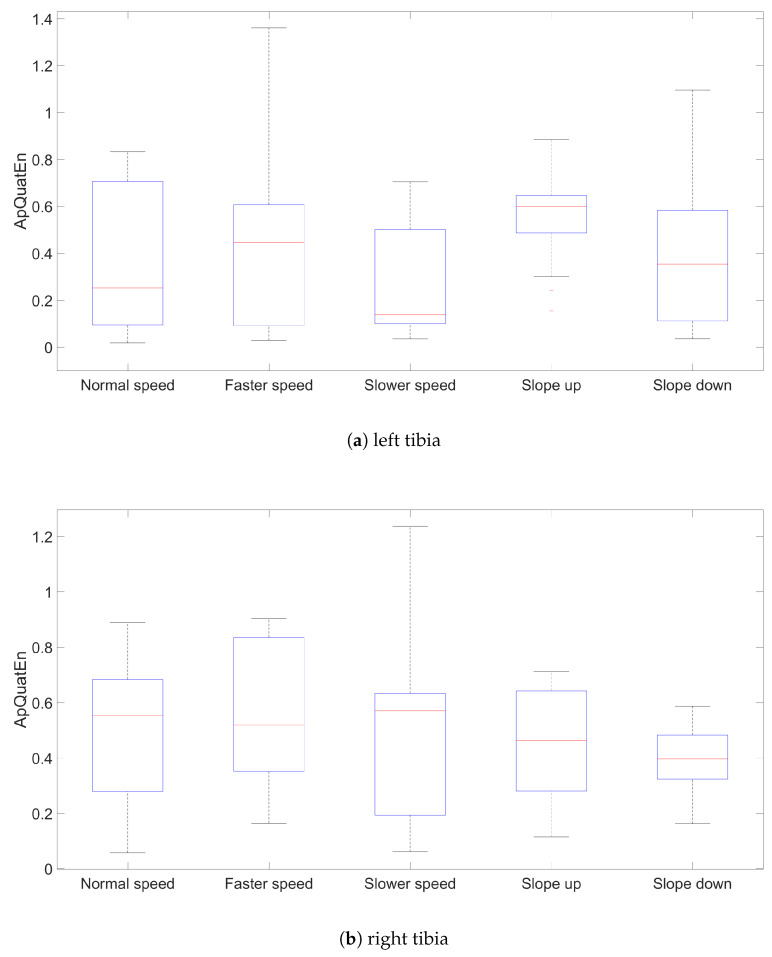
Results values of ApQuatEn (m=2, $r=\mathrm{mean}(d_{\mathrm{cosine}})$) for left and right tibia segments.

**Figure 4:**
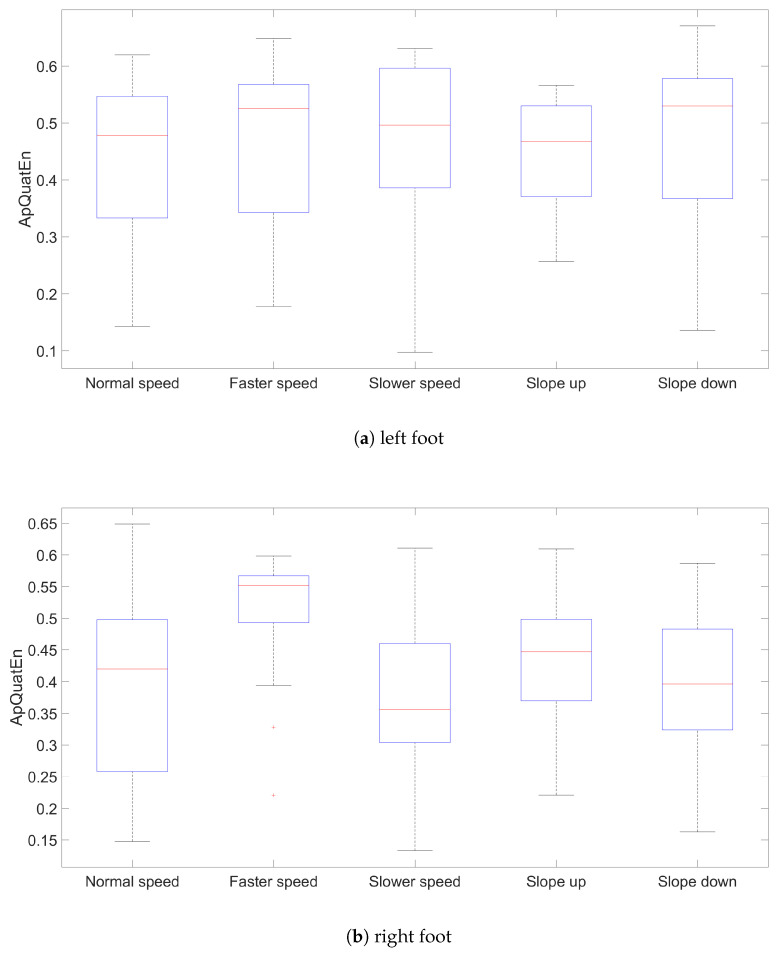
Results values of ApQuatEn (m=2, $r=\mathrm{mean}(d_{\mathrm{cosine}})$) for left and right foot segments.

**Figure 5:**
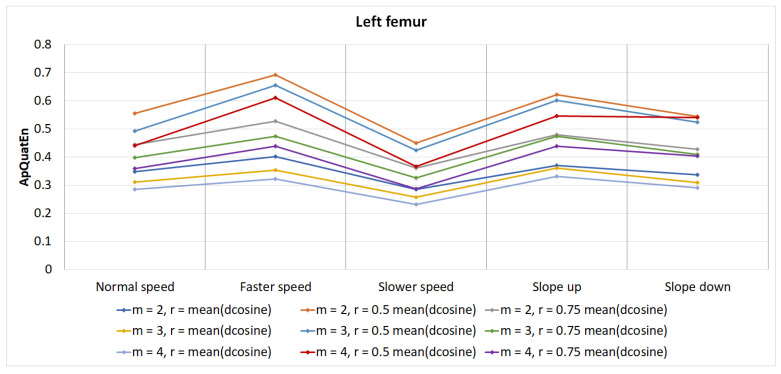
The value of entropy ApQuatEn in relation to the length of vector (*m*) and threshold distance *r* value for left femur segments.

The results for ApQuatEn (m=2, $r=\mathrm{mean}(d_{\mathrm{cosine}})$) are presented using box plots comprising all three segments for left and right side, speed and slope (Figure 2, Figure 3 and Figure 4). The other parameters’ configurations give results presenting similar dependencies. The influence of parameters to result in quaternion approximate entropy is presented in Figure 5. Movements of all considered body parts and experiments configurations are characterized by the positive value of entropy, which detect and quantify chaotic behavior. This is consistent with results obtained with use of LLE in [22], for which all times series are characterized by positive values of LLE. The median values for all segments are also presented in Table 1. In addition, the aggregated median values for segments without taking into account the side of the body are included.

The values of entropy are the smallest for *Slower* speed for segments femur (median 0.286), tibia (median 0.233) and foot (median 0.410), which can indicate more regularity and predictability movement in those segments [16]. The highest values for different speed configurations are for all segments in *Faster* speed configuration. The values of entropy for different treadmill inclinations (*Up* and *Down*), where participants maintain normal speed, are somewhat

Higher than for configuration with *Normal* speed and flat treadmill. Differences of entropy values between configurations with treadmill inclinations for different segments are somewhat higher for *Up* than *Down* configuration. Big differences between entropy values for left and right tibia segments for *Slower* speed are interesting. Generally, values for femur segments (median value: *Normal* 0.337, *Faster* 0.388, *Up* 0.387, *Down* 0.371) are smaller than for other segments, which can indicate more regularity in movement in this segment. The exception is *Slower* speed configuration where the smallest value is for tibia segments (median 0.233).

In [22], the two variants of LLE, based on quaternion angle, were estimated: the short-term LLE for the first stride and the long-term LLE over a fixed interval between the fourth and the tenth stride. The Pearson correlation coefficient for values of ApQuatEn (m=2, $r=\mathrm{mean}(d_{\mathrm{cosine}})$) for left and right femurs throughout all experiment configurations is 0.735 for the long-term LLE and 0.709 for the short-term LLE.

Table 2, Table 3 and Table 4 present the Pearson correlation coefficient of ApQuatEn (m=2, $r=\mathrm{mean}(d_{\mathrm{cosine}})$) in each segment and configuration. We can observe that values of proposed quaternion approximate entropy are characteristics for two groups of experiment configurations—treadmill velocity (*Normal* and *Faster*) and slope (*Up* and *Down*). All Pearson correlation coefficients in those two groups for all segments are greater than 0.5. The Pearson correlation coefficients are greater for *Slower* and *Up*, *Slower* and *Down* than for *Slower* and *Normal*, *Slower* and *Faster* for femur and foot segments. This dependency is not so significant for tibia segments. The gaits with *Normal* and *Faster* speed have similar properties in the context of chaotic behaviors. The same can be observed for gaits on an inclined treadmill at slope *Up* and *Down*.

The detailed influence of lengths of vector *m* and value of threshold *r* into result ApQuatEn for left femur segments is presented in Figure 5. All values of entropy have similar dependencies within experiment configurations and segments. Generally, the ApQuatEn for m=4 is smaller than for m=2. This means that differences between $\Phi^{4}\left(r\right)$ and $\Phi^{5}\left(r\right)$ are smaller than $\Phi^{2}\left(r\right)$ and $\Phi^{3}\left(r\right)$. Typically, it is suggested that, for clinical data, *m* is to be set at 2 when utilizing the ApEn algorithm [7].

**Figure 6:**
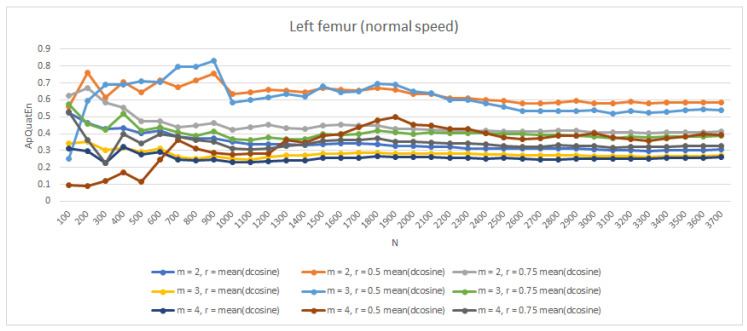
The value of entropy for left femur segments (*Normal* speed) ApQuatEn (m=2, $r=\mathrm{mean}(d_{\mathrm{cosine}})$) in relation to data length *N*.

In [14], ApEn values were quantified on generated data from 100 to 10,000 data points, in increments of 100. As can be seen, the entropy values stabilize around *N* of 2000. In order to check the stabilization of entropy ApQuatEn values as N increased in experiment data, the chaotic logistic map was subjected to entropy analysis up to an *N* of 3700 data points. For this particular analysis, (m=2, $r=\mathrm{mean}(d_{\mathrm{cosine}})$) was chosen. Entropy was quantified on data in increments of 100. As can be seen in Figure 6, the entropy values stabilize around an *N* of 2700 time samples. For gait with *Normal* velocity, this is after a twenty-second stride.

## 4. Conclusions

This publication concerns the new quaternion approximate entropy ApQuatEn for quaternion time series represented orientations of human skeleton segments. The presented approach was used to quantify chaotic behavior in the context of regularity in time series. The example of application for treadmill walking data, in five variants of walking speed and treadmill slope, was discussed. Data was obtained from the optical motion capture system. It was confirmed that all considered time series are characterized by positive values of entropy values which quantify a local instability. The use of entropy as a mathematical tool to quantify predictability of gait parameters is an entirely emerging task in human movement research. This is the first proposition to consider quaternion signal and has analysed the correlated data which describe rotation in 3D. A possible future work can be related to develop quaternion version of negentropies to develop hypothesis testing for normality [32].

## Figures and Tables

**Table 1:** Median values of ApQuatEn (m=2, $r=\mathrm{mean}(d_{\mathrm{cosine}})$).

	Normal	Faster	Slower	Up	Down
lfemur	0.334	0.371	0.274	0.405	0.344
rfemur	0.345	0.406	0.300	0.371	0.396
**femur**	**0.337**	**0.388**	**0.286**	**0.387**	**0.371**
ltibia	0.252	0.446	0.138	0.599	0.353
rtibia	0.554	0.519	0.570	0.463	0.396
**tibia**	**0.338**	**0.519**	**0.233**	**0.537**	**0.385**
lfoot	0.478	0.525	0.496	0.467	0.529
rfoot	0.420	0.552	0.356	0.447	0.396
**foot**	**0.443**	**0.544**	**0.410**	**0.447**	**0.447**

**Table 2:** The Pearson correlation coefficient of ApQuatEn (m=2, $r=\mathrm{mean}(d_{\mathrm{cosine}})$) calculated for left and right femur segments.

Left Femur						Right Femur					
	Normal	Faster	Slower	Up	Down		Normal	Faster	Slower	Up	Down
**Normal**	1.000	0.699	0.378	0.157	0.334	**Normal**	1.000	0.862	0.412	0.347	0.343
**Faster**	0.699	1.000	0.462	0.303	0.476	**Faster**	0.862	1.000	0.699	0.537	0.604
**Slower**	0.378	0.462	1.000	0.921	0.941	**Slower**	0.412	0.699	1.000	0.899	0.951
**Up**	0.157	0.303	0.921	1.000	0.944	**Up**	0.347	0.537	0.899	1.000	0.834
**Down**	0.334	0.476	0.940	0.944	1.000	**Down**	0.343	0.604	0.951	0.834	1.000

**Table 3:** The Pearson correlation coefficient of ApQuatEn (m=2, $r=\mathrm{mean}(d_{\mathrm{cosine}})$) calculated for left and right tibia segments.

Left Tibia						Right Tibia					
	Normal	Faster	Slower	Up	Down		Normal	Faster	Slower	Up	Down
**Normal**	1.000	0.695	0.733	0.143	0.564	**Normal**	1.000	0.611	0.718	0.874	0.458
**Faster**	0.695	1.000	0.632	0.328	0.549	**Faster**	0.611	1.000	0.505	0.496	0.439
**Slower**	0.733	0.632	1.000	0.409	0.744	**Slower**	0.718	0.505	1.000	0.797	0.685
**Up**	0.143	0.328	0.409	1.000	0.542	**Up**	0.874	0.496	0.797	1.000	0.646
**Down**	0.564	0.549	0.744	0.542	1.000	**Down**	0.458	0.439	0.685	0.646	1.000

**Table 4:** The Pearson correlation coefficient of ApQuatEn (m=2, $r=\mathrm{mean}(d_{\mathrm{cosine}})$) calculated for left and right foot segments.

Left Foot						Right Foot					
	Normal	Faster	Slower	Up	Down		Normal	Faster	Slower	Up	Down
**Normal**	1.000	0.728	0.510	0.439	0.491	**Normal**	1.000	0.721	0.491	0.135	0.390
**Faster**	0.728	1.000	0.778	0.573	0.773	**Faster**	0.721	1.000	0.449	0.241	0.237
**Slower**	0.510	0.778	1.000	0.837	0.923	**Slower**	0.491	0.449	1.000	0.901	0.909
**Up**	0.439	0.573	0.837	1.000	0.751	**Up**	0.135	0.241	0.901	1.000	0.841
**Down**	0.491	0.773	0.923	0.751	1.000	**Down**	0.390	0.237	0.909	0.841	1.000
